# Targeting Cancers with oHSV-Based Oncolytic Viral Immunotherapy

**DOI:** 10.3390/cimb46060334

**Published:** 2024-06-03

**Authors:** Rakin Tammam Nasar, Ifeanyi Kingsley Uche, Konstantin G. Kousoulas

**Affiliations:** 1 School of Medicine, Louisiana State University Health Sciences Center, New Orleans, LA 70112, USA; rnasar@lsuhsc.edu; 2 Division of Biotechnology and Molecular Medicine, Department of Pathobiological Sciences, School of Veterinary Medicine, Louisiana State University, Baton Rouge, LA 70803, USA

**Keywords:** herpes, oncolytic viruses, immunotherapy, HSV

## Abstract

The recent success of cancer immunotherapies, such as immune checkpoint inhibitor (ICIs), monoclonal antibodies (mAbs), cancer vaccines, and adoptive cellular therapies (ACTs), has revolutionized traditional cancer treatment. However, these immunotherapeutic modalities have variable efficacies, and many of them exhibit adverse effects. Oncolytic viral Immunotherapy (OViT), whereby viruses are used to directly or indirectly induce anti-cancer immune responses, is emerging as a novel immunotherapy for treating patients with different types of cancer. The herpes simplex virus type-1 (HSV-1) possesses many characteristics that inform its use as an effective OViT agents and remains a leading candidate. Its recent clinical success resulted in the Food and Drug Administration (FDA) approval of Talimogene laherparevec (T-VEC or Imlygic) in 2015 for the treatment of advanced melanoma. In this review, we discuss recent advances in the development of oncolytic HSV-1-based OViTs, their anti-tumor mechanism of action, and efficacy data from recent clinical trials. We envision this knowledge may be used to inform the rational design and application of future oHSV in cancer treatment.

## 1. Introduction

Cancer remains a leading cause of death worldwide. According to the World Health Organization (WHO), the global cancer burden was estimated to account for nearly 10 million deaths in 2020, and this burden continues to increase [1]. Current anti-cancer drugs and immunotherapies have variable efficacy and unwanted adverse effects [2,3,4]. These shortcomings emphasize the urgency of developing newer and more efficacious therapies against several cancers.

Oncolytic viral immunotherapy (OViT) represents a novel cancer treatment modality in which oncolytic viruses (OVs) are used to infect and destroy cancer cells. The oncolytic lysis of tumor cells releases tumor associated antigens (TAAs) and provides an immunogenic context that triggers the activation of anti-tumor immune responses [5,6,7]. OVs also represent an attractive combination partner with other traditional and modern cancer immunotherapies, particularly with immune checkpoint inhibitors (ICIs) [8,9].

To date, only four OVs have been approved globally for treatment in patients with cancer, and they include: (a) an unmodified picornavirus called Rigvir (ECHO-7), which was approved in 2004, in Latvia, for treatment in patients with melanoma [10]; (b) Oncorine (H101), a modified adenovirus that achieved approval in China in 2005 for treatment of head and neck cancer [11]; (c) Talimogene laherparevec (T-VEC or Imlygic), an attenuated herpes simplex virus (HSV-1) approved in 2015 in the United States [12]; and (d) triple-mutated HSV-1 called Delytact (Teserpaturev/G47∆), recently approved in Japan in 2021, for the treatment of malignant glioma [10]. Although, there are other OVs that are being currently studied and tested in preclinical animal models and clinical trials, and each has key properties that inform their utility [13,14]. Due to the extensive clinical studies that have culminated in the primacy of an oncolytic herpes simplex virus (oHSV) to become the first OV to be approved by the Food and Drug Administration (FDA), we focus this review primarily on oHSV-based OViT.

HSV-1 possess many characteristics that inform its use as a preferred OViT agent. These include the incredible amount of knowledge of the molecular biology of the virus, its ability to infect a variety of cell types, its large genome (152 kb) that allows for insertion of additional transgenes, its relative safety, and its inability to cause insertional mutagenesis [9,15]. oHSV-based OViT gained considerable attention after the approval of T-VEC in 2015. In this review, we discuss recent advances in the development of oncolytic HSV-1-based OViTs, their anti-tumor mechanism of action, and efficacy data from recent clinical trials. We envision this knowledge may be used to inform the rational design and application of future oHSV in cancer treatment.

## 2. Mechanism of oHSV Anti-Tumor Activity

oHSV mediates its anti-tumor efficacy through multiple mechanisms. These mechanisms include direct tumor lysis, alteration of the tumor microenvironment (TME), recruitment of tumor infiltrating lymphocytes, and priming of immune responses mediated by innate and tumor-specific adaptive immune cells [9,16,17,18].

**Figure 1:**
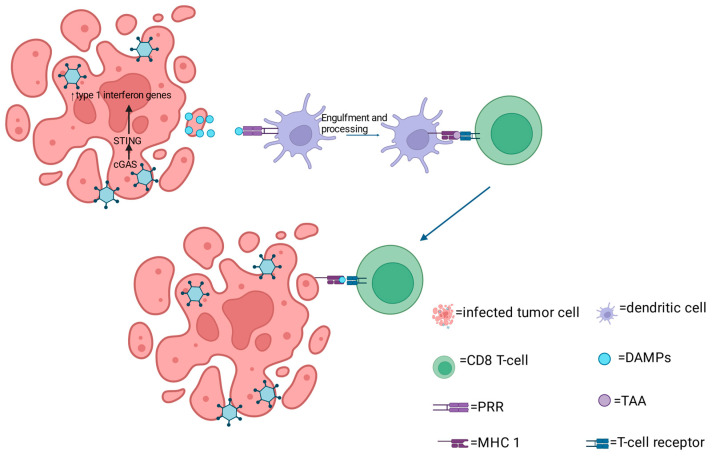
oHSV mechanism of antitumor activity. oHSV mediated tumor lysis leads to the release of TAAs which get recognized by PRRs located on APCs, particularly dendritic cells. These dendritic cells then presents the TAAs via MHC class 1 to CD8+ T cells which results in their activation and recruitment into the tumor bed where the recognize and target tumor cells expressing similar TAAs.

Following oHSV infection, tumor cells undergo lysis and immunogenic cell death (ICD). It has been shown that oHSV-induced ICD of both melanoma and squamous cell carcinoma cells resulted in the release of damage-associated molecular patterns (DAMPs) such as ATP and high mobility group protein B1 (HMGB1) in vitro [19,20]. In addition, the viral infection of these cancer cells promoted the translocation of calreticulin (CRT) to the cell surface [19,20]. The exposure of these DAMPs is thought to serve as a “find me” signal, which interacts with pattern recognition receptors (PRRs) on innate cells, particularly immature dendritic cells (DCs) (Figure 1). These recruited DCs become matured, phagocytose dead cells, and initiate adaptive anti-tumor immunity by cross-presenting TAAs to CD8+ T-cells.

Another mechanism used by the host immune system to sense cancer is through the cyclic guanosine monophosphate–adenosine monophosphate synthase (cGAS) and stimulatory of interferon genes (STING) complex. cGAS is an essential cytosolic DNA sensor that responds to genotoxic cell stress by binding to abnormal DNA in neoplastic cells, which then activates downstream adaptor molecule, STING [20,21]. Upon activation, STING initiates the transcription of type I interferon genes, and production of inflammatory cytokines [20,21]. The cGAS-STING signaling pathway has also been implicated in the priming of anti-tumor T-cell response and the trafficking of immune cells to the TME [22].

On the other hand, the activation of the cGAS-STING signaling pathway may be detrimental for the spread and replication of the virus within the tumor. Data evaluating the opposite contributions of the STING signaling pathway in cancer cell lysis and tumor immunogenicity are currently limited. Although, Guendalina et al. have shown that STING-knockout tumors infected with oHSV supported the virus spread and its lytic potential but showed an impaired ICD and exhibited molecular signatures of an immunosuppressive TME [23]. While enormous efforts have been made to understand the critical role of cGAS-STING in the immune system, details of its role in anti-tumor immunity are not fully understood, and further studies were warranted.

Efficient eradication of tumors involves a cyclic process called cancer immunity cycle [24]. To become tumor-specific CD8+ T-cells, naïve CD8+ T-cells in the draining lymph nodes are presented with TAAs in the context of the major histocompatibility complex (MHC) class I molecules by professional antigen-presenting cells (APCs), particularly DCs [25]. Further priming of naïve CD8+ T-cells is influenced by cytokine production and costimulatory signals from the APCs, which drives their conversion to an effector state [26]. These activated CD8+ T-cells undergo massive clonal expansion and then they traffic to and infiltrate the tumor, where they recognize TAAs and eliminate cancer cells. The killing of cancer cells then releases additional TAAs which begins another cycle.

The CD8+ T-cells’ mediated response is an essential part of the adaptive anti-tumor immunity for controlling tumors [17,26]. Using a mouse B16F10 melanoma model, Ifeanyi et al. showed that the therapeutic efficacy of a vaccine strain oHSV, VC2, was dependent on CD8+ T-cells lymphocytes. This is corroborated by the evidence that the loss of CD8+ T-cells by in vivo treatment with anti-CD8 monoclonal antibodies in immunocompetent mice, impaired the tumor clearance and survival benefits provided by VC2 therapy [17]. Results from other research groups have also echoed the importance of CD8+ T-cells anti-tumor immunity induced by oHSV-OViT in preclinical mouse models of other cancer types [27,28]. Also, VC2 was shown to produce significant antitumor activity against breast cancer cells using the 4T1/Balb/c immunocompetent and syngeneic mouse model. Specifically, intra-tumor treatment reduced lung metastasis associated with increased T cell infiltration comprised of CD4+ and CD4+CD8+ double-positive T cells. Characterization of purified tumor infiltrating T cells, associated with reduction of pro-tumor PD-L1 and VEGF gene transcription, improved T cell responses and reduce pro-tumor biomarker gene transcription [29]. Recently, the anti-tumor immunotherapeutic potential of VC2 was significantly increased by the constitutive expression of GM-CSF. This new VC2-GM-CSF virus produced drastic reduction in 4T1 cell metastasis to the lungs of Balb/c mice after a single intratumoral administration (Nabi, Kousoulas, submitted). VC2 holds promise for further development as an oncolytic and immunotherapeutic approach to treat melanoma, breast and other cancers.

Studies that assessed the levels of effector cytotoxic T lymphocytes (CTLs) in the TME showed that elevated CD8+ T-cells in TME was a good factor in predicting the efficacy and prognosis of patients with various cancers [26,30]. Although in some tumors, the immunosuppressive milieu of the TME, which constitute cold tumors, can pose as a barrier and suppress the tumor trafficking of CD8+ T-cells and their effector functions [31]. OViT offers the potential to overcome this shortcoming, which is usually observed with other cancer treatment modalities like ICIs. For example, when oHSV HSV1716 was used as a combinatory therapy with a monoclonal antibody that blocks program death-1 (PD-1) checkpoint in a mouse model of rhabdomyosarcoma, it resulted to an improved survival and increased tumor infiltration of CD4+ and CD8+ T-cells, but not of regulatory T-cells (Tregs), compared to the PD-1 blockade or HSV1716 treatments alone [32]. 

Further, efficacy data from a phase 1b clinical trial [NCT02263508] in 21 patients with advanced melanoma demonstrated that treatment with anti-PD-1 antibody (pembrolizumab) combined with T-VEC resulted to an increased tumor infiltration of IFN-γ+ CD8+ T-cells in patients with tumors with low level of immune cell infiltrates and negative IFN-γ+ signatures [8]. Importantly, a high objective response rate (ORR) of 62% with a 33% complete response was observed for the combination therapy compared with previous treatments with pembrolizumab alone [8]. These studies highlights the importance of CD8+ T-cells as the chief commanders of anti-tumor immunity and the backbone of cancer immunotherapy. In addition, they suggest that Ovs, particularly oHSV-derived OVTs, can modify the TME to be more susceptible to checkpoint inhibitors in order to promote CD8+ T-cells’ anti-tumor immunity.

## 3. oHSV in Preclinical Models and Clinical Trials (Table 1)

### 3.1. Talimogene Laherparevec (T-VEC or Imlygic)

**Figure 2:**
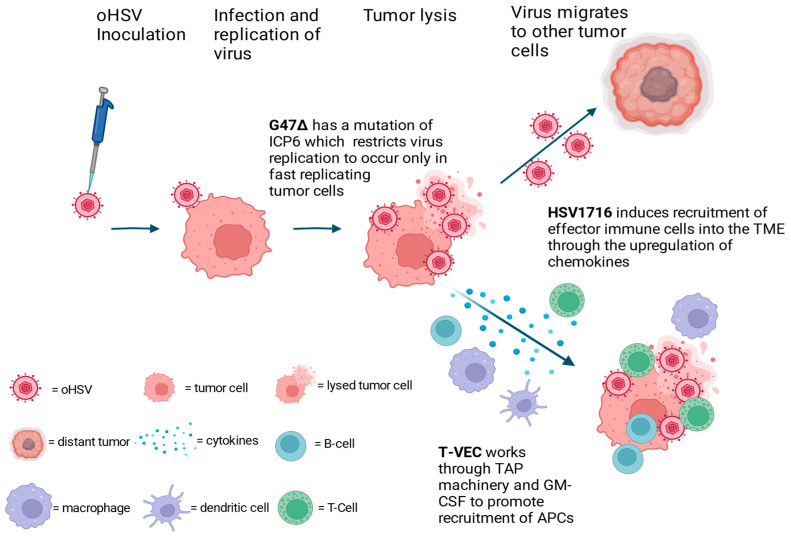
Herpes derived oncolytic virotherapy. Each oHSV is engineered to possess unique properties that enhance their oncolytic activities. oHSVs infect and replicate within the tumor cells. Tumor lysis of infected tumor leads to the release of chemokines, which aid in the recruitment of immune cells. Recruited immunologic cells become activated and target tumor cells. Viral spread allows for oHSVs infection and lyses of distant tumor cells.

T-VEC is the first OVT approved by the FDA for treatment of advanced unresectable melanoma [12]. This virus is a replication-competent HSV-1 JS-1 strain, which is modified to contain deletions of both infected cell protein 34.5 (also known as neurovirulence factor ICP34.5) and ICP47 genes [33]. These deletions limit the virus replication in cancer cells and enhances antigen presentation through the transporter associated with antigen processing (TAP) machinery [9,34]. Additionally, the virus expresses the human granulocyte–macrophage colony stimulating factor (GM-CSF), which functions to promote the recruitment of APCs, as shown in Figure 2 [33].

T-VEC demonstrated significant anti-tumor effects when tested in vitro in human cancer cell lines and in vivo in mice. When administered into A20 lymphoma tumors in mice, the virus improved tumor shrinkage and clearance [33]. There were evidence that intratumoral treatment with virus induced a systemic immune response to distal, untreated tumors, a phenomenon known as the abscopal effect [33]. The incorporation of GM-CSF was thought to be the reason for the improved tumor reduction in distal, untreated tumors. Although, the data shown in this study do not indicate any significant difference in the reduced tumor size of the contralateral non-treated tumors in mice that received the viruses with or without GM-CSF. Thus, it is not entirely clear what or how GM-CSF contribute to the systemic immune response that cascades to distal, uninjected tumors. Also, there are currently no data showing that the virus directly lyse distal tumors. Preclinical evidence suggest that the virus uses immune-mediated mechanism of action, particularly tumor specific CD8+ T-cell response as its main mode of systemic efficacy [35].

Early phase I studies evaluating the safety profile of T-VEC demonstrated that the virus was safe and well tolerated following intratumoral injection in patients with melanoma [36]. The main side effects reported included low-grade fever, chills, and injection site reactions [36] (Table 1). A phase II trial in 50 patients with stage IIIC to IV melanoma showed that intralesional administration of the virus resulted to an ORR of 26%, with responses observed in both injected and uninjected lesion, including visceral lesions [37] (Table 1). Biopsy of regressing lesions from patients treated with T-VEC had increased MART-1 (melanoma antigen recognized by T-cells 1) specific CD8+ T-cell infiltration and a reduction in the levels of suppressor cells such as CD4+FoxP3+ Treg cells [37]. Subsequent randomized phase III studies that utilized the virus as a monotherapy for patients with unresected stage IIIB to IV melanoma yielded durable response rates (DRR) of 16.3%, and an ORR of 26.4%, compared to patients who received recombinant GM-CSF with DRR and ORR of 2.1% and 5.7%, respectively. Further, T-VEC provided a significant overall survival (OS) benefit to treated patients compared to GM-CSF therapy [37]. This study was pivotal in the approval of T-VEC monotherapy for patients with advanced melanoma.

The efficacy of combining T-VEC with other immunotherapies such as checkpoint blockade have been evaluated in preclinical models and have paved the way for this emerging treatment paradigm in humans [35]. When the virus was used as a combinatory therapy with an antibody that blocks T-cell checkpoint inhibitory receptors such as PD-1, or cytotoxic T lymphocyte antigen 4 (CTLA-4), promising results were achieved in treated patients [8,38,39].

Interestingly, a recent phase III, randomized, double-blind study [NCT02263508] showed that there was no significant benefit for the combination of T-VEC and pembrolizumab over placebo plus pembrolizumab in patients with unresectable stage IIIB/IVM1c melanoma after a median of 31 months of follow-up [40]. The T-VEC + pembrolizumab group had an ORR of 48.6% compared to 41.3% for the placebo + pembrolizumab group, which was not statistically significant. In addition, there was no statistical significance in the median OS between the treatment groups. T-VEC + pembrolizumab combination had a favorable safety profile, which was comparable to that of pembrolizumab alone [40] (Table 1). It is not entirely clear why no benefit was achieved in this study. A potential factor may be in the design of the treatment regimen. The conflicting mechanisms of the cGAS-STING pathway might also explain why no additional benefit was seen in this study. In a previous phase 1b study (already discussed in thex previous section) that precluded this study, pembrolizumab was administered after two doses of T-VEC. This strategy allows for seroconversion of HSV-naïve patients and may have prevented rapid clearance of the virus by an enhanced anti-viral immune response mediated by pembrolizumab. Finding an optimal balance between anti-viral immune response and anti-tumor response is critical for OViT mediated control of tumor progression. However, in this study, both T-VEC and pembrolizumab were administered simultaneously. Also, a successful combinatory therapeutic outcome may be dependent on the stage or progression of the disease being treated. More studies are needed to define the optimal therapeutic conditions of T-VEC OViT when used in monotherapy or in combination with therapeutic agents.

Despite the great progress that led to its approval, T-VEC still faces many challenges, such as how it is administered and dosed. The virus is administered as a local injection directly to cutaneous, subcutaneous, or nodal sites, and not all patients have injectable disease. Other limitations of the virus usage include its low response rate and its effect on distant and visceral metastases [41]. Although T-VEC was initially approved as an adjuvant therapy, several studies aimed to evaluate the benefits of the virus in a neoadjuvant setting have shown promising results, and more studies are underway [41].

Further, while T-VEC have shown great success in melanoma treatment, this begs the question of whether it is effective against other cancer types like ovarian, rectal, and pancreatic cancer. To this end, several groups are currently investigating the potential application of T-VEC as a more generalized cancer therapy [42].

### 3.2. Delytact (Teserpaturev or G47∆)

Delytact is the first OV approved for treatment of brain cancer. Delytact is a triple-mutated replication-competent OV based on the HSV-1 (F) strain [10]. This virus was developed by adding another mutation, i.e., the deletion of the gene encoding ICP47, to the genome of parental G207 virus, which contains deletions in both copies of the gene encoding for ICP34.5 and an inactivation of the gene encoding for ICP6 [10]. The ICP6 gene encodes for a subunit of ribonucleotide reductase, an enzyme important for nucleotide metabolism and viral DNA replication in quiescent cells [43]. The inactivation of the ICP6 gene further restricts the virus to selectively replicate in tumor cells.

Several works have shown G47Δ replicated and lyse in a variety of cancer cell lines. In vitro experiments that compared the cytocidal activity of G47Δ to G207 in multiple cancer cell lines, including glioma cancer cells, showed that G47Δ replicated better and killed the tumor cells more rapidly than its parental virus [44]. Like in T-VEC, the gene encoding for ICP47 in G47Δ is mutated. HSV-1 uses the ICP47 gene product to inhibit the TAP machinery [34]. Consequently, G47Δ restored MHC class I expression in infected human melanoma cells [44]. These G47Δ-infected tumor cells stimulated T-cells to a greater extent than G207-infected cells and secreted more IFN-γ [44]. Thus, the deletion of the ICP47 gene increases antigen presentation and enhances the immunogenicity of oHSVs that possess the mutation.

In an in vivo setting, treatment with G47Δ was superior at reducing tumor growth rate and enhancing survival of mice transplanted with glioma cancer cells. When the virus (2 × 10^6^ plaque-forming units [PFU]) was intracerebrally administered to A/J strain mice, which are known to be susceptible to HSV-1, each treated mouse survived for 3 weeks without any remarkable symptoms, whereas all mice treated with wild-type HSV-1 (2 × 10^3^ PFU) deteriorated within 7 days [44]. Also, all mice infected with G207 (2 × 10^6^ PFU) survived [44]. This result indicates that G47Δ has an enhanced anti-tumor efficacy, but retained a good safety profile that is comparable with G207. The anti-tumor efficacy of G47Δ has since been tested in mouse models of prostate cancer [45], nasopharyngeal carcinoma [46], breast cancer [47], esophageal carcinoma [48] hepatocellular carcinoma [49], schwannoma [50], and malignant peripheral nerve sheath tumor [51]. These studies form the basis for clinical translation of G47Δ in humans.

The first-in-human clinical trial [UMIN000002661] in patients with recurrent/progressive glioblastoma (GBM) demonstrated that G47Δ was safe when inoculated into the brain of humans [52] (Table 1). Each patient underwent two intratumoral injections (at a dose of 1 × 10^9^ PFU) within 2 weeks. No evidence of viral shedding was obtained in the blood, urine, and saliva, by quantitative polymerase chain reaction (PCR). Histology analysis showed increased infiltration of CD4+ and CD8+ T-cells in biopsy samples obtained after first injection, compared to pre-G47∆ administration. The median OS observed was 30.5 months from initial surgery (initial diagnosis) and 7.3 months from the last G47∆ administration [52]. The authors also reported that three patients out of the 13 patients in study survived longer than 46 months.

Building upon the described study above, a phase II study [UMIN000015995] was conducted in 19 patients with residual or recurrent GBM [53] (Table 1). In this single-arm study, each patient received 1 × 10^9^ PFU of G47∆ intratumorally for a total of six doses at 5–14 days for the first two doses and 4 ± 2 weeks for subsequent doses. The virus was well tolerated, and the main side effects reported were fever, nausea, vomiting, and lymphopenia. Biopsy histology confirmed the presence of a larger number of tumor-infiltrating CD4+ and CD8+ T-cells and persistent low numbers of Foxp3+ cells following repeated G47∆ treatments [53]. The median OS of patient after receiving G47Δ treatments was 20.2 months, which far exceeded the data of previous treatments. In addition, five patients survived longer than 3 years after receiving G47∆ treatments. This study was the grounds for the conditional approval of G47∆ by the Japan Ministry of Health, Labor, and Welfare (MHLW).

Current treatment options for recurrent GBM include re-operation, checkpoint inhibitors, systemic chemotherapy, and/or bevacizumab, which all have limited efficacy [54]. The median OS with these treatments does not exceed 5–10 months, which is far from the readout of G47Δ therapy. The results obtained from G47Δ treatment against recurrent GBM appear promising and warrant further investigation in phase 3 clinical trials. It will also be interesting to investigate the synergetic effect of G47Δ therapy combined with checkpoint blockades for GBM patients. Beyond GBM, G47Δ has also been evaluated in patients with castration-resistant prostate cancer [UMIN000010463], olfactory neuroblastoma [jRCTs033180325] and malignant pleural mesothelioma [jRCTs033180326].

### 3.3. HSV1716 (Seprehvir)

HSV1716 is derived from the HSV-1 strain 17 and possesses a deletion in the gene encoding the neurovirulence factor ICP34.5 [55]. Studies in mouse models of 4T1 mammary carcinoma demonstrated that HSV1716 therapy moderately reduced the number of metastases in the lungs of treated group compared to those that received mock treatment [56]. In addition, HSV1716 treated mice that survived initial challenge rejected a second 4T1 tumor re-challenge. Immunohistochemical analyses revealed the presence of CD4+ and CD8+ T-cells in tumors treated with the virus, whereas very few inflammatory cells were found in the mock-treated tumors. HSV1716 was ineffective in reducing tumor growth and metastases in SCID mice, which are deficient in both T-cells and B-cells [56]. This supports the author’s in vitro experiment, which indicates that HSV1716 does not replicate well in 4T1 tumors cells. Overall, this results suggest that the virus needs an intact adaptive immune response to control tumor growth. 

HSV1716 OV promotes the recruitment of effector immune cells into the TME through the upregulation of chemokines: monokine induced by IFN-γ (MIG also known as CXCL9) and IFN-γ inducible protein-10 (IP-10 also known as CXCL10) [57]. These chemokines are known to have potent chemotactic activities and predominantly target activated T-cells and natural killer (NK) cells. Other intratumoral immune alterations in mouse studies that have been reported to be associated with HSV1716 therapy include the reduction of immunosuppressive FoxP3+ Tregs and the polarization of macrophages from pro-tumoral M2 towards anti-tumoral M1 [58].

The safety of HSV1716 has been evaluated in several clinical trials for melanoma, mesothelioma, and high-grade glioma (HGG) [59,60]. Preliminary studies in five patients with stage IV metastatic melanoma demonstrated that the virus was well tolerated when it was administered intratumorally(at a dose of 10^3 ^PFU) [59] (Table 1). Pathological examination of excised nodules showed evidence of tumor necrosis in three patients that received two or more doses of HSV1716. Additionally, immunohistochemical staining of injected nodules showed that viral replication was restrained to tumor cells and not adjacent normal tissues.

In 2000, Rampling et al. reported the first study evaluating the safety HSV1716 as potential treatment for patients with GBM. Nine patients were enrolled in the study and were divided into three cohorts (three patients per cohort), with each receiving 10^3^, 10^4^ and 10^5^ PFUs of HSV1716, respectively, by stereotactic injection [60]. No adverse clinical symptoms or latent viral reactivation due to HSV1716 administration was reported. Subsequent clinical studies where the virus was injected either directly into tumor bed or resected tumor sites in GBM patients further echoed the safety and efficacy of HSV1716.

**Table 1:** Referenced clinical trials that use oHSVs.

Name	Strain and Mutation	Mechanism	Clinical Trials	Patient Group	Major Findings	Reference
T-VEC	HSV-1 JS-1 strain, which is modified to contain deletions of both infected cell protein 34.55 (also known as neurovirulence factor ICP34.5) and ICP47 genes.	Enhances antigen presentation through the TAP machinery. The virus expresses GM-CSF, which functions to promote the recruitment of APCs.	Phase I	30 patients with Stage IIIC or IV melanoma	The virus was safe and well tolerated following intratumoral injection in patients with melanoma.	[36]
			Phase II NCT00289016	50 patients with stage IIIC to IV melanoma	Intralesional administration of the virus resulted to an ORR of 26%, with responses observed in both injected and uninjected lesion, including visceral lesions.	[37]
			Phase III NCT00769704a	436 patients with unresected stage IIIB to IV melanoma	T-VEC provided a significant OS benefit to treated patients compared to GM-CSF therapy.	[12]
			Phase III NCT02263508	692 patients with unresectable stage IIIB/IVM1c melanoma	No significant difference in ORR between T-VEC + pembrolizumab vs. placebo + pembrolizumab. No significant difference in safety profile of T-VEC + pembrolizumab vs. placebo + pembrolizumab.	[40]
G47∆	HSV-1 F strain, which contains deletion of the gene encoding ICP47, to the genome of parental G207 virus, which contains deletions in both copies of the gene encoding for ICP34.5 and an inactivation of the gene encoding for ICP6.	The gene which encodes ICP6 which isa subunit of ribonucleotide reductase, an enzyme important for nucleotide metabolism and viral DNA replication in quiescent cells [42]. The inactivation of the gene encoding for ICP6 further restricts the virus to selectively replicate in tumor cells.	Phase I UMIN000002661	13 patients with Recurrent/progressive GBM	G47Δ was safe when inoculated into the brain of a human.	[52]
			Phase II UMIN00001 5995	19 patients with residual or recurrent GBM	The virus was well tolerated, and the main side effects reported were fever, nausea, vomiting, and lymphopenia. Biopsy histology confirmed the presence of a larger number of tumor-infiltrating CD4+ and CD8+ T-cells and persistent low numbers of FoxP3+ cells following repeated G47∆ treatments.	[53]
			Phase I UMIN000010463	castration-resistant prostate cancer	G47∆ safety has been examined in other cancers.	[52]
			Phase I jRCTs033180325	olfactory neuroblastoma	G47∆ safety has been examined in other cancers.	[52]
			Phase I jRCTs033180326	malignant pleural mesothelioma	G47∆ safety has been examined in other cancers.	[52]
HSV1716	HSV-1 strain 17, whichpossesses a deletion in the gene encoding the neurovirulence factor ICP34.5	HSV1716 OV promotes the recruitment of effector immune cells into the TME through the upregulation of chemokines: monokines induced by IFN-γ (MIG also known as CXCL9) and IFN-γ inducible protein-10 (IP-10 also known as CXCL10).	Pilot study	five patients with stage IV metastatic melanoma	Immunohistochemical staining of injected nodules showed that viral replication was restrained to tumor cells.	[59]
			Phase I	Nine patients with GBM	No adverse clinical symptoms or latent viral reactivation due to HSV1716 administration.	[60]

## 4. Discussion

oHSV is a promising therapy that has been demonstrated to be safe and efficacious in both preclinical and clinical settings in the treatment of melanoma, GBM, mesothelioma and HGG. oHSV directly lyse tumor cells, which, subsequently, release TAAs and provide the microenvironment for anti-tumor responses including infiltration of immune cells, particularly CD8+ T-cells, which are important players in the anti-tumor immune response. The release of DAMPs recruits DCs to initiate adaptive anti-tumor immunity to present the TAAs to CD8+ T-cells. The cGAS-STING pathway is thought to play a role in anti-tumor immunity; however, there is conflicting evidence about the exact nature of that role. On the one hand, cGAS-STING is thought to be involved in priming T-cells and directing immune cells to the TME [20,21,22]. On the other hand, STING-knockout tumors infected with oHSV showed more efficient viral spread and lysis, but less expression of TAAs at the TME [23].

CD8+ T-cell infiltration, as well as infiltration of other T-cells, plays an important role in mediating the therapeutic efficacy of oHSV. This has been demonstrated in preclinical studies in which impaired T-cell immunity showed worse tumor clearance and survival in a B16F10 melanoma mouse model [17]. This is the rationale behind why using a combinatory therapy with ICIs would be an effective approach in maximizing therapeutic efficacy of oHSVs [8,26,27,28,29,30]. Further, phase III clinical trials validating the findings of the combinatory efficacy of oHSV are imperative, especially in the cases of T-VEC and G47∆, where phase II and phase III clinical trials have already been conducted. The results of a phase III, randomized, double blind study using T-VEC and pembrolizumab did not show increased treatment efficacy compared to pembrolizumab alone in patients with unresectable stage IIIB/IVM1c melanoma [40]. There may have been insufficient expression of TAAs at the TME that prevented adequate immune infiltration at the site of the tumor. Further, clinical trials using PD-1 inhibitors may demonstrate promising results considering the previous results of T-VEC used in combination with a CTLA-4 inhibitor [8,38,39]. It is imperative to expand the study of T-VEC to other stages of melanoma as well to use it as a combinatory treatment with PD-1 inhibitors or CTLA-4 inhibitors. Similarly, combinatory therapy, with oHSV and checkpoint inhibitors, could have favorable efficacy in patients with recurrent GBM, considering their efficacy in a phase I and II studies [52,53].

It is important to ensure that oHSV mediated lysis is limited to tumor cells and not to normal tissue, or else patients may suffer unintended side effects, or the treatment may have reduced efficacy. In the case of HSV1716, it has been proven to be safe in various cancers including melanoma, mesothelioma, HGG, and GBM. It was also shown to only replicate within injected nodules in a study with patients with stage IV metastatic melanoma [59,60]. Similar safety outcomes have been reported for T-VEC and G47Δ. T-VEC with pembrolizumab was shown to have a safety profile similar to that of pembrolizumab alone in a phase III, randomized, double-blind study in patients with unresectable stage IIIB/IVM1c melanoma [40]. In the case of G47Δ against GBM, there was no quantification found through PCR of the virus spreading to the blood, urine, or saliva. In another study, side effects of G47Δ were reported, but they were well tolerated. The side effects include fever, nausea, vomiting, and lymphopenia [53].

Pathological and immunological markers have shown that oHSVs can cause changes associated with enhanced anti-tumor activity. In an early phase I study of T-VEC, biopsy of injected and uninjected lesions showed regression and had increased MART-1 specific CD8+ T-cell infiltration, and a reduction in suppressor cells [37]. Similar results of increased CD4+ and CD8+ infiltration were shown histologically in a clinical trial using G47Δ in patients with GBM [52]. The immune response shown using these two oHSVs further supports the evidence that oHSV induces robust antitumor immunity. Also, the abscopal effect observed in these studies suggests that the induced antitumor immune response specifically targets tumor cells expressing similar tumor markers regardless of the site or method of OV inoculation. This demonstrates that oHSV could potentially be used in late stages of other diseases where the tumor may have metastasized to other regions.

## 5. Conclusions

oHSVs have been shown be safe and efficacious in preclinical and clinical studies. However, much more needs to be done to fully understand how the immune response can be optimized to maximize the therapeutic efficacy of oHSV therapies. There are several ongoing clinical studies testing the efficacy of different oHSVs against different cancers including mammary carcinoma, prostate cancer, nasopharyngeal carcinoma, breast cancer, esophageal carcinoma, hepatocellular carcinoma, schwannoma, and malignant peripheral nerve sheath tumors. However, there are barriers to the utilization of oHSV therapies in other types of cancers especially the metastatic ones. Optimizing an efficient delivery system is a major issue. Intratumoral inoculation is the common route of treatment that has been reported in several preclinical and clinical studies. This method allows for the OVs to by bypass blood dilution, host immune antiviral immunity, and prevent non-targeted cell injuries. Utilizing an intravenous route of OV delivery is an ideal method of treatment as it allows for broad virus distribution, which can infect both primary and metastatic tumors, regardless of location. Tumor heterogeneity is another issue that may decrease the efficacy of the oHSV activities. Nevertheless, the application of oHSV therapies continue to show promising results in the treatment of metastatic tumors. For example, the FDA recently approved the oHSV, MVR-T3011 for the treatment of recurrent or metastatic head and neck squamous cell cancer. MVR-T3011 was reported to be effective in reducing the size of tumors distant from initial injection site. Further, there is a growing body of evidence that indicates that oHSVs would be effective if used in combination with ICIs. However, more studies need to be done to identify the proper treatment combinations, dosing amount, or dosing schedule for treating various cancer types. Overall, oHSVs is a bonafide immunotherapy that possess a safe and efficacious profile that can be used to treat different cancers.

## Data Availability

The original contributions presented in the study are included in the article, further inquiries can be directed to the corresponding authors.
